# Gastrointestinal stromal tumor in Carney’s triad with laparoscopic total gastrectomy: a case report

**DOI:** 10.1186/s40792-024-02041-2

**Published:** 2024-10-23

**Authors:** Hajime Midoritani, Hironori Kawada, Kosuke Kaneda, Shuichiro Toda, Kento Awane, Keisuke Tanino, Kaichiro Harada, Keigo Tachibana, Masahiko Honjo, Koji Kitamura, Mami Yoshitomi, Yoshiharu Shirakata, Ryuta Nishitai

**Affiliations:** Hyogo Amagasaki General Center, Amagasaki, Japan

**Keywords:** Carney’s triad, Gastrointestinal stromal tumors (GISTs), Laparoscopic total gastrectomy

## Abstract

**Introduction:**

Carney's triad is a rare syndrome characterized by the co-occurrence of gastric gastrointestinal stromal tumor (GIST), pulmonary chondroma, and extra-adrenal paraganglioma. We present a case of a young woman with GISTs associated with this triad.

**Case presentation:**

A 28-year-old woman was identified with multiple gastric tumors and a right lung nodule during a routine health check-up. CT scans and upper gastrointestinal endoscopy revealed a 50 mm mass on the lesser curvature of the stomach, along with two additional gastric lesions and a 20 mm nodule in the right lung. The patient had a history of right middle lobectomy at the age of 19 for pulmonary chondroma. During surgery, enlarged lymph nodes were observed, indicating metastasis, which necessitated a total gastrectomy with radical (D2) lymph node dissection. Pathological examination confirmed seven GISTs, with immunohistochemical staining positive for KIT (+), DOG1 (+), and negative for SDHB (−). The postoperative course was uneventful, and the patient was discharged on the seventh postoperative day. Despite opting out of adjuvant imatinib therapy, she remains disease-free 2 years postoperatively.

**Conclusions:**

This case underscores the necessity of total gastrectomy with lymph node dissection due to the high incidence of metastasis in GISTs associated with Carney's triad. Further research is required to determine the optimal extent of lymph node dissection in such cases.

## Background

Carney's triad is a non-hereditary syndrome first proposed by Carney in 1977, characterized by the co-occurrence of at least two out of three specific tumors: gastric gastrointestinal stromal tumor (GIST), pulmonary chondroma, and extra-adrenal paraganglioma [1]. GISTs associated with Carney's triad exhibit distinct characteristics compared to typical GISTs. Herein, we report a case of a patient with GIST associated with Carney's triad who underwent laparoscopic total gastrectomy, along with a review of the relevant literature.

## Case presentation

**Fig. 1:**
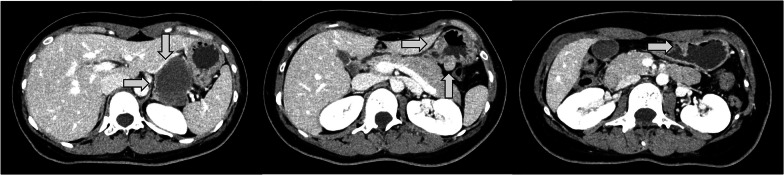
Preoperative CT; there were four multiple gastric masses (white arrows)

**Fig. 2:**
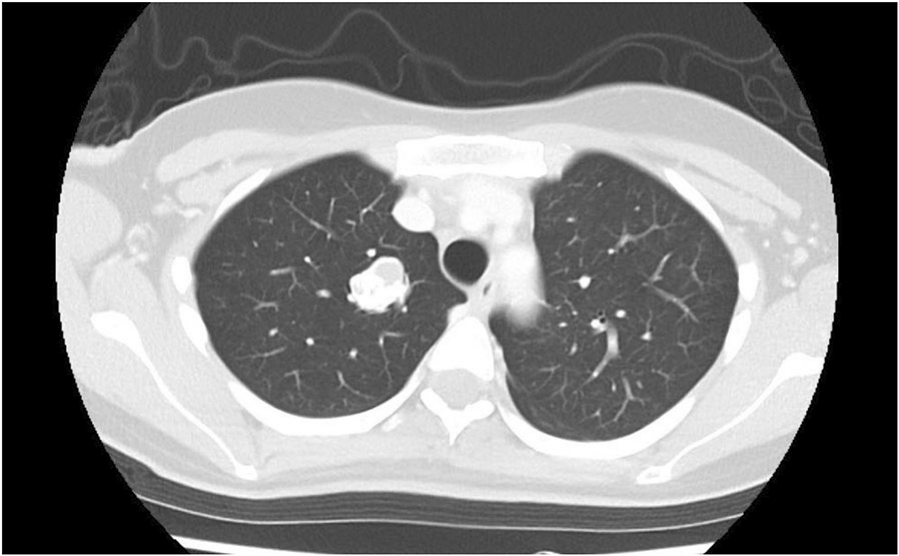
Chest plain CT; there was a nodule with coarse calcifications in the upper lobe of the right lung

**Fig. 3:**
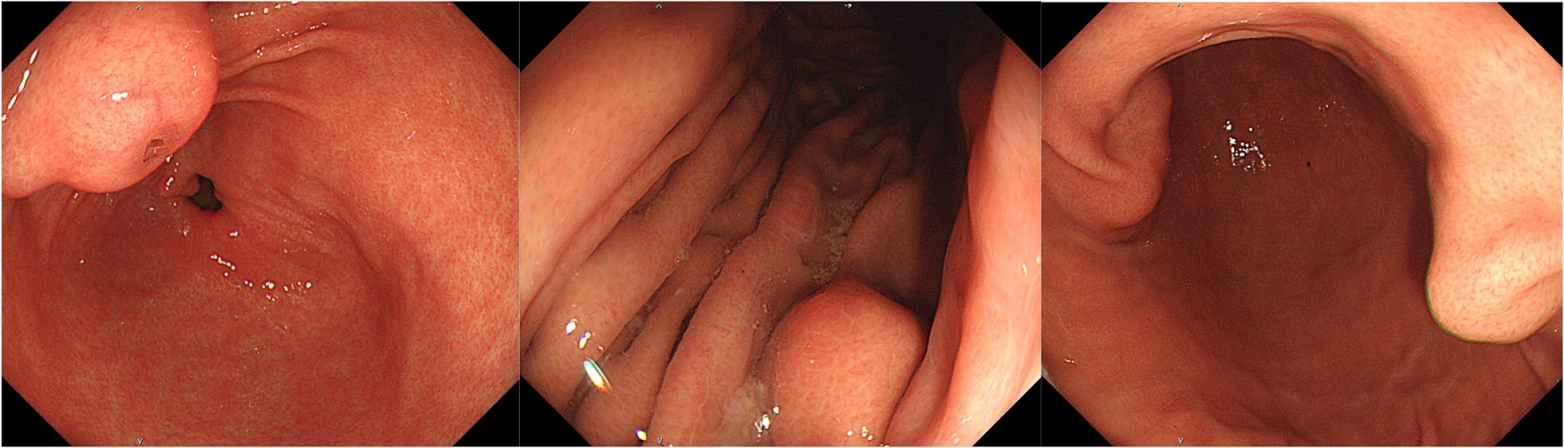
Endoscopic examination; there were multiple submucosal tumors in the stomach

A 28-year-old woman was found to have a nodule in the upper right lung field during a chest X-ray examination conducted as part of a health check-up. She was referred to our hospital for further evaluation. She had no specific complaints, and her physical examination was unremarkable. There was no significant family history. Blood tests were normal, and tumor markers were not elevated. Her medical history included a right middle lobectomy performed at the age of 19 for pulmonary chondroma. A computed tomography scan revealed a 50 mm mass on the lesser curvature of the upper body of the stomach and two other tumor-like lesions in different locations (Fig. 1). A 20 mm nodule with coarse calcifications was observed in the upper lobe of the right lung (Fig. 2). An endoscopic examination showed a 25 mm submucosal tumor on the anterior wall of the gastric angle, a 15 mm submucosal tumor on the posterior wall of the lower body of the stomach, and a 13 mm submucosal tumor on the greater curvature of the upper body of the stomach (Fig. 3).

Based on the above findings, the diagnosis was multiple GISTs associated with Carney's triad. Since it is rare for lung tumors to occur repeatedly in a young woman with simultaneous presence of multiple GISTs in the stomach, it was concluded that the lung nodule is likely a pulmonary chondroma associated with Carney’s triad, and its biopsy was not performed. Considering the possibility that the gastric tumor-like lesions could be extra-adrenal paragangliomas, an I123-metaiodobenzylguanidine scintigraphy was performed. However, there was no uptake in the tumor-like lesions, and both blood and urine catecholamine levels were negative, leading to the exclusion of this possibility.

**Fig. 4:**
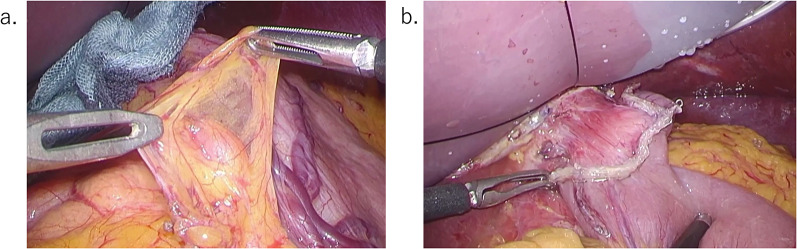
Intraoperative findings; **a** There was an enlarged lymph node on the lesser curvature, and rapid pathological examination revealed it to be metastasis of GIST. **b** We changed the surgery plan from a local resection to a total gastrectomy with radical lymph node dissection with Roux-en-Y reconstruction

We planned a laparoscopic local resection of the stomach for the multiple gastric GISTs to preserve gastric function. During surgery, an enlarged lymph node was observed on the lesser curvature which was not detected by preoperative CT, and rapid intraoperative pathological examination indicated lymph node metastasis of the GIST (Fig. 4). The detection of lymph node metastasis necessitated a systematic lymph node dissection. Therefore, the surgical procedure was changed to a total gastrectomy with radical lymph node dissection. The surgery lasted 6 h and 16 min, with a blood loss of 50 ml.

**Fig. 5:**
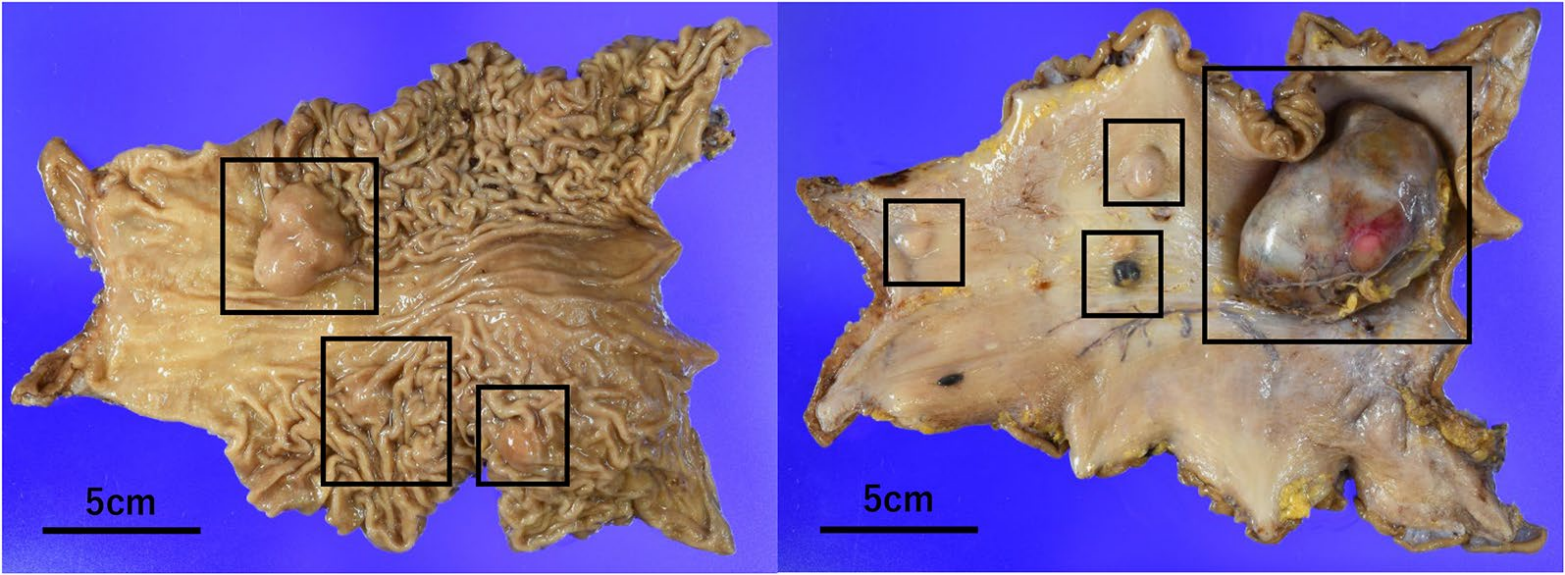
Pathological examination; there were three submucosal tumors that were not detected preoperatively, totaling seven lesions (black squares)

**Fig. 6:**
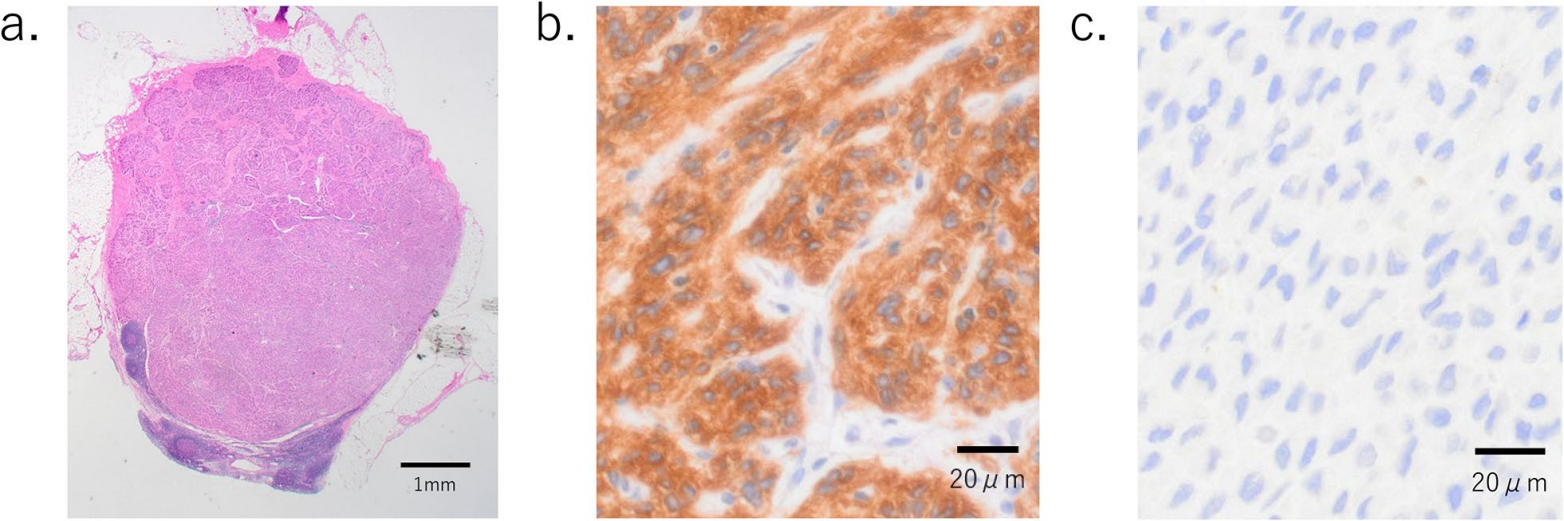
Pathological stainings; **a** hematoxylin eosin staining of the #3 lymph node removed during surgery, **b** immunoreactivity with KIT antibodies. **c** Tumors were succinate dehydrogenase B deficient

Pathological examination revealed multiple lesions smaller than 10 mm that had not been detected during preoperative testing, and a total of seven tumors were identified (Fig. 5). Immunohistochemical staining showed KIT (+), DOG1 (+), and Succinate dehydrogenase B (SDHB) (−) (Fig. 6), confirming the diagnosis of GIST associated with Carney's triad. The mitotic count was 1 per 50 HPF, and the tumor was classified as intermediate risk according to the Modified Fletcher criteria. The lymph nodes submitted during surgery showed infiltration of KIT-positive tumor cells, confirming metastasis of the GIST. No metastasis was found in the other dissected lymph nodes.

The postoperative course was uneventful, and the patient was discharged on the seventh postoperative day. Although adjuvant imatinib therapy was considered, it was not administered at the patient's request. As of 2 years postoperatively, the patient has been progressing without recurrence of the gastric lesions. The pulmonary chondroma has been followed-up to monitor for any increase in tumor size or development of respiratory symptoms.

## Discussion

Carney's triad is characterized by three specific tumors: gastric leiomyosarcoma, pulmonary chondroma, and extra-adrenal paraganglioma [1]. The complete type involves all three tumors, while the incomplete type includes any two, typically involving gastric leiomyosarcoma. The complete type is rare, accounting for approximately 20% of cases, with the majority being the incomplete type. It has been reported that Carney’s triad has an average survival of 26.5 years from the initial gastric resection, and due to its slow progression, the prognosis is considered to be good. The average interval from the first to the second tumor has been reported to be 8.4 years, and from the second to the third to be 5.9 years [2], indicating the need for long-term follow-up.

Typical GISTs are most commonly found in individuals aged 60–70 years, with no significant gender difference [3]. In contrast, gastric GISTs associated with Carney's triad predominantly occur in females aged 10–20 years [2]. When GISTs are identified in young women, it is crucial to consider the possibility of Carney's triad and implement long-term follow-up.

Typical GISTs have a low incidence of lymphatic metastasis, with only 3% exhibiting lymph node metastasis according to a study of 200 cases by DeMatteo et al. [4]. In contrast, GISTs associated with Carney's triad frequently exhibit lymph node metastasis and multicentric occurrence [2]. Zhang et al. reported a 29% incidence of lymph node metastasis in 104 cases of Carney's triad [5], which is significantly higher than in typical GISTs. Although metastases are often reported on the lesser curvature, specific sites of lymph node metastasis have not been well documented. Currently, there are no comprehensive reports summarizing the locations of lymph node metastasis specific to Carney's triad.

In typical GISTs, a study of 20 cases in Japan reported that more than half of the lymph node metastases occurred on the lesser curvature, specifically involving nodes #1 and #3 [6]. In this case, the #3 lymph node was confirmed to be metastasis. According to the Japanese guideline [7], systematic lymph node dissection is not recommended due to its unclear benefits. However, in cases where young women present with GISTs, intraoperative observation of the lesser curvature lymph nodes is advised because of the potential association with Carney’s triad. If enlargement is noted, rapid pathological examination should be considered to confirm metastasis. When positive, further investigation is needed to determine the necessity of systematic lymph node dissection. Considering the 30% incidence of lymph node metastasis in Carney's triad, at least D1 dissection should be considered.

The Japanese guideline recommend local resection for GISTs to preserve organ function [7]. However, considering that Carney's triad is characterized by multiple lesions confined to the stomach and a high rate of lymph node metastasis, it is important to be prepared to perform total gastrectomy in cases associated with Carney's triad. According to Zhang et al.'s report of 104 cases, total gastrectomy was performed in only 20% of initial surgeries. Among the 80% of cases that did not receive total gastrectomy, 70% required additional resections due to recurrence in the remaining stomach. There were also cases requiring up to eight resections, and ultimately 45% of the 104 cases underwent total gastrectomy [5]. In this case, postoperative specimen revealed lesions not detected in preoperative examinations, suggesting that stomach preserving surgery may end up residual tumors and the need for additional resections.

In Carney's triad, GISTs are often c-KIT negative, making them resistant to imatinib [8]. In this case, imatinib was not administered postoperatively at the patient's request. Currently, there are no effective chemotherapies for gastric GISTs associated with Carney's triad, which further underscores the need to prioritize radical surgical approaches during the initial operation.

## Conclusion

We experienced a case of a young woman with incomplete Carney's triad presenting with GISTs. Due to the detection of lymph node metastasis during intraoperative rapid examination, laparoscopic total gastrectomy was performed. Gastric GISTs associated with Carney's triad exhibit distinct characteristics compared to typical gastric GISTs, and surgical approach and follow-up strategies should be carefully considered.

## Data Availability

None.

## Abbreviations

GIST: Gastrointestinal stromal tumor
SDHB: Succinate dehydrogenase B
